# Experimental Protocol for Color Difference Evaluation Under Stabilized LED Light

**DOI:** 10.3390/jimaging11010004

**Published:** 2024-12-30

**Authors:** Sofiane Vernet, Eric Dinet, Alain Trémeau, Philippe Colantoni

**Affiliations:** Laboratoire Hubert Curien, UMR 5516, 18 rue Benoît Lauras, 42000 Saint-Etienne, France; sofiane.vernet@univ-st-etienne.fr (S.V.); eric.dinet@univ-st-etienne.fr (E.D.); alain.tremeau@univ-st-etienne.fr (A.T.)

**Keywords:** color gamut, printer characterization, perceptible color differences, psychophysical experiments, LED panel characterisation, LED stabilization

## Abstract

There are two key factors to consider before implementing a color discrimination experiment. First, a set of color patches should be selected or designed for the specific purpose of the experiment to be carried out. Second, the lighting conditions should be controlled to eliminate the impact of lighting instability on the experiment. This paper addresses both of these challenges. It proposes a method to print pairs of color patches with non-noticeable color differences. It also proposes a method to stabilize the Spectral Power Distributions (SPDs) of a Light-Emitting Diode (LED) lighting system. Finally, it introduces an experimental protocol for a color discrimination study that will be performed thanks to the contributions presented in this paper.

## 1. Introduction

Research on human vision relies heavily on psychophysical experiments, which serve as both its most crucial tool and its greatest limitation. Designing, preparing, and conducting such experiments are often tedious and time-consuming tasks. Moreover, it is frequently challenging to create an experiment that precisely aligns with the specific needs of the research topic under investigation. For instance, most experiments on color vision use samples from existing color systems (e.g., Pantone, NCS, or Munsell). While these systems provide excellent color consistency, they impose constraints on the range of experimental parameters available.

The present study, which is an extended version of a conference paper published in 2024 in [1], is part of a broader effort to optimize white light for individuals with low vision. It uses selected pairs of closely related color samples to evaluate from a color assessment experiment how effectively a particular light source enables color discrimination.

Many methods of color assessment exist in the literature. The most popular and effective approaches are based on gray scale [2,3] and color difference pairs [4] with or without separation [5,6], color matching [7], magnitude estimation [8] or hue cancellation techniques [9]. We chose to use the paired comparison method since it was more adapted to the problem of studying color discrimination for low-vision people [10]. To achieve that, we needed to obtain a large amount of low color difference pairs of color patches. Early in the design process, it became evident that commercially available sets of color samples did not offer colors with sufficient proximity, requiring the creation of a custom set. To address this, we employed the inkjet printing technology to produce closely related color samples. However, using a commercial inkjet printer introduced several challenges, including the calibration and prediction of color output, the production of closely related color pairs, and the control of printed samples.

The choice of the lighting system is another critical component in psychophysical experiments particularly when the task involves pairs of color samples with subtle differences. For instance, LED panels often exhibit fluctuations in output due to heat buildup during operation [11,12,13] and aging of components [14]. In the literature, LEDs have been studied in order to model their behavior as a function of their junction temperature. Chhajed et al. [15] measured the junction temperature of four types of LEDs and reviewed their optical properties at different temperatures. They showed that increasing the temperature of LEDs induced a noticeable difference in light rendering. In order to fix such an issue, various approaches have been proposed. For example, Qu et al. [16] used the measurement of junction temperature to stabilize the output of an RGB LED lamp. Chen et al. [17] proposed a model using optical, thermal and electrical parameters. Li et al. [18] integrated photodetectors into the LEDs when Llenas et al. [19] integrated a compact spectroradiometer to stabilize the output of a tunable LED device using a closed-loop feedback system. Additionally, Hofbauer et al. [20] designed a controller to minimize fluctuations in the illumination of an LED solar simulator. As an alternative approach, we introduced in [1] an algorithm that dynamically adjusts the drive levels of the individual channels of a multispectral LED lighting unit to stabilize the output throughout the duration of an experiment.

The goal of the study presented in this paper is to propose an experimental protocol that integrates the production of customized color samples via inkjet printing with the stabilization of LED lighting. This approach ensures that psychophysical experiments on low vision, planned for the next phase of this research, will meet the specific requirements of color discrimination studies while achieving reliable repeatability.

The color management process of the inkjet printer detailed in Section 2 is a new contribution. Similarly, the technical protocol introduced in Section 4 is also a new contribution. Conversely, the characterization of the LED lighting system summarized in Section 3 was introduced in [1]. A critical analysis of these methods is given in Section 5. Next, a conclusion is drawn in Section 6 summarizing the main contributions of this paper. The proposed methods will enable color discrimination experiments to be conducted with visually impaired people and the optimization of LED lighting systems to improve color vision for both visually impaired and normally sighted people.

## 2. Methods Used for the Selection of Color Pairs

To conduct a psychophysical experiment on color vision, one approach involves using pairs of color samples [4,5,21], each consisting of two color patches with small color differences.

Two main options can be considered: either using pre-existing standard color patches (e.g., Pantone, Munsell Book of Color, RAL colors) or creating custom patches using a colorimetrically calibrated printer. The first option provides only limited control over the characteristics of the patches, making it less flexible for specific experimental needs. The second option, more suitable for experiments on color vision, involves printing custom color patches with an inkjet printer designed for high-quality photographic printing. For our study, we selected the Canon image PROGRAF PRO-300, which uses 10 color ink cartridges. The patches were printed on matte paper, which is an ideal choice for the goals of a color discrimination experiment.

**Figure 1:**
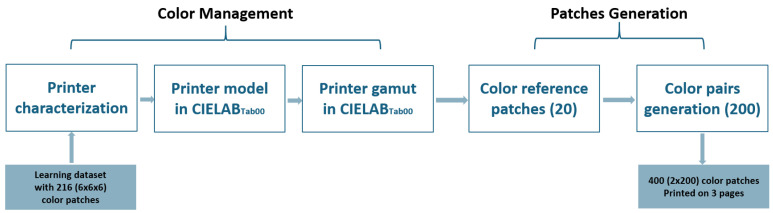
The different steps of the color workflow used to print color pairs.

In the following subsections, we detail the new method we propose for sampling the printer’s color gamut to generate *N* reference color patches from a selected sampling grid where all neighbors of an element of the grid are equidistant for a chosen color distance formula. We also introduce an additional method designed to select pairs of color samples that are equidistant and colorimetrically close (see Figure 1). For both methods, we used the printer’s native RGB color space to produce the targeted color patches. This approach bypasses the default color management of the operating system, giving us more precise control over the printed samples.

### 2.1. Sampling Cielab Color Space with Ciede*00* Metrics

The CIEDE00 (${\Delta E}_{00}$) metric is widely considered as one of the most accurate methods for assessing just noticeable color differences [22]. Even if other metrics have been introduced recently [5], we used it to evaluate small color differences between color patches. CIEDE00 is a color difference formula specifically designed to account for the non-uniformity of the CIELAB color space in regard to lightness, chroma and hue differences [23]. However, one main limitation of the CIEDE00 metric is its lack of Euclidean structure [22,24,25]. This limitation complicates its application in some computational tasks, such as uniform sampling within a color space, where it is essential to generate color patches that are equidistant from one another in a colorimetric sense based on a defined sampling grid.

Sampling based on non-Euclidean Color Difference Formulas (CDFs) presents numerous challenges [26,27]. Colantoni et al. proposed a robust algorithmic method in [28] to sample the CIELAB color space with a non-Euclidean CDF allowing the use of the Euclidean metric for a chosen CDF. This approach relies on a tabulated sampling of the CIELAB color space derived from the CIEDE00 distance (the corresponding color values for this sampling are available at https://www.couleur.org/deltaE/d00.h (accessed on 29 December 2024)), which is used to define the base color values of the patches required to carry out the psychovisual experience described in Section 4.

**Figure 2:**
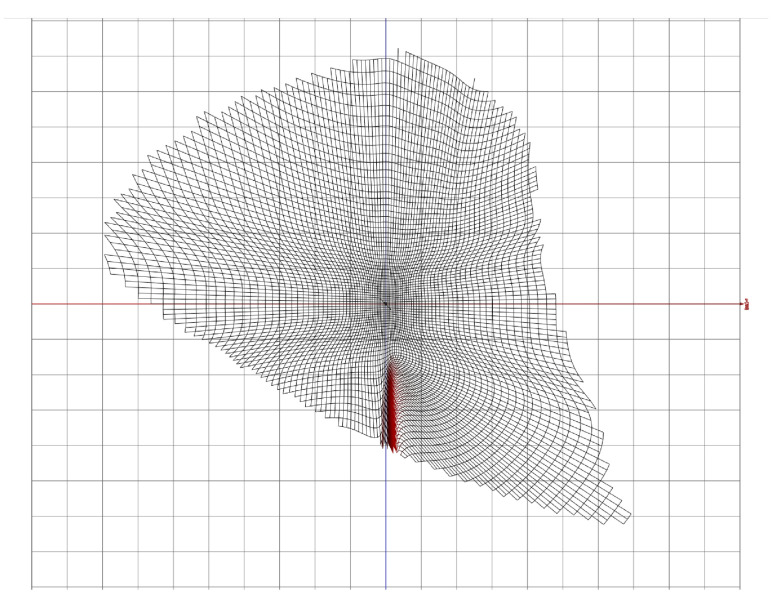
The distance is here set to 1 to visualize a slice of the $CIELAB_{Tab00}$ tabulated values in the chromatic plane (a∗,b∗) of the CIELAB color space.

The sampling forms a cubic grid of size (152×321×312). Each element of the grid corresponds to a CIELAB color (defined by its (L∗,a∗,b∗) coordinates), ensuring that the CIEDE00 distance from its 6 neighbors is equal to 0.5. This grid is centered on the color coordinate (50,0,0) and serves as the basis for a new tabulated color space named $CIELAB_{Tab00}$. Figure 2 illustrates the distribution of the tabulated color values in the chromatic diagram (a∗,b∗), highlighting a red zone where calculated values are sub-optimal. For further details on the accuracy of this sampling, refer to [28].

To convert color values from this $CIELAB_{Tab00}$ tabulated color space into (L∗,a∗,b∗) coordinates, we use the values from the grid combined with a trilinear interpolation.

For the inverse transformation from (L∗,a∗,b∗) coordinates to tabulated $CIELAB_{Tab00}$ color values, we construct a tetrahedral structure from the original sampling grid by dividing each cube into 6 tetrahedra. When converting a (L∗,a∗,b∗) color, we first identify the tetrahedron containing the color and then apply tetrahedral interpolation to calculate the corresponding $CIELAB_{Tab00}$ tabulated color value.

### 2.2. Color Management

Printer color management techniques rely on either traditional methods such as those described in [29] or machine learning approaches [30,31]. These techniques use standardized color profiles, mainly ICC and iccMAX profiles [32,33], or direct measurements of pre-printed color patches. Their goal is to maximize the colorimetric capabilities of the device and, most importantly, ensure consistent color reproduction across various devices.

As stated at the beginning of Section 2, we chose to work within the printer’s RGB color space. This RGB color space is influenced not only by the printer hardware but also by the color management system implemented by the operating system driving the printer (in this case, Windows 10). To link the RGB values used to control the printing of selected color patches (i.e., those defined by the tabulated color space $CIELAB_{Tab00}$) to the measurements derived from their printing, it is essential to implement a color management system that is both accurate and straightforward. To achieve this, we propose a method originally designed for characterizing and calibrating monitors.

#### 2.2.1. Printer Characterization and Color Profiles

**Figure 3:**
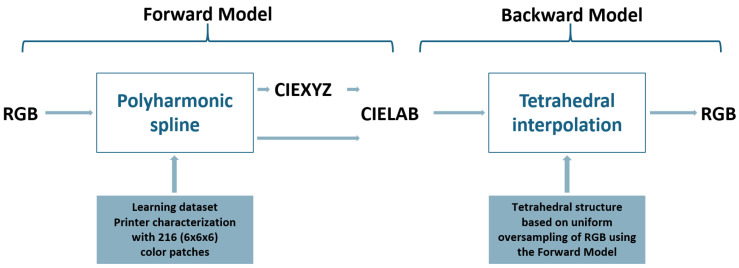
Forward and backward models.

**Figure 4:**
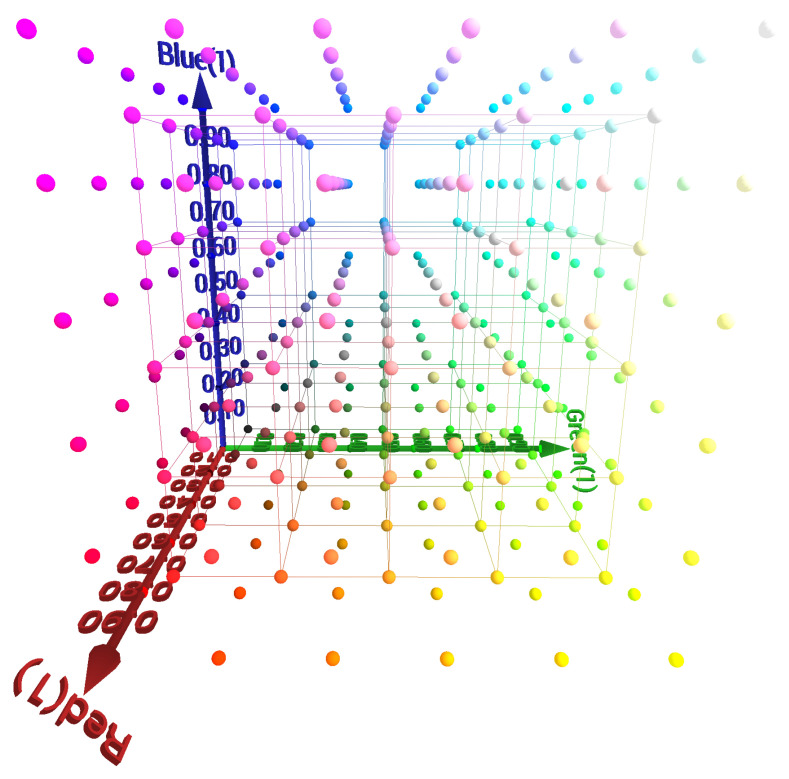
RGB color space discretization with 216+125 patches. The 125 patches correspond to color samples linked by segments.

To characterize the printer, we used the color management process introduced in [34]. This robust method, originally designed for displays, is essential for achieving accurate cross-media color reproduction. The technique introduced aimed to predict displayed color stimuli from given RGB inputs using polyharmonic spline interpolation for the forward direction (RBG to CIELAB) and tetrahedral interpolation for the backward direction (CIELAB to RGB) (see Figure 3). This approach relied on an optimally optimized set of color patches measured on a screen, enhancing accuracy without technology-specific assumptions. The forward model mapped RGB inputs to the CIELAB color space, while the backward model found RGB values for specific CIELAB targets. An iterative selection of color patches improves the training data set, thereby refining model precision across various displays. However, since the dynamic selection of color patches is not possible for printers, we opted for a straightforward discretization of the RGB space. For the training set, we used 216 patches arranged in a (6×6×6) grid. For the test set, we used 125 patches in a (5×5×5) grid (see Figure 4).

After printing the 216+125 patches on matte paper, we measured them using a spectrocolorimeter (I1 Pro 3 from xRite) and used the resulting reflectance factors to obtain the CIEXYZ and CIELAB values under illuminant D65, which was chosen as the reference illuminant. Based on the 125 test patches, we estimated the optimal parameters for the forward model (also used for the backward model): the polyharmonic kernel (linear, cubic or thin-plate), the target space (CIELAB or CIEXYZ) and the smoothing factor.

Our forward model uses CIELAB as the default target. This does not imply that we have to use this space as the target for the polyharmonic kernel. In fact, we considered two choices. We can use either CIELAB, which seems to be the most logical target, or CIEXYZ associated with a (X,Y,Z) to (L∗,a∗,b∗) color transformation. The use of different color spaces as targets gives us another degree of freedom. We estimated the forward model for the three possible kernels and the two target color spaces (CIEXYZ and CIELAB) across a range of smoothing factor values. The model that minimizes the mean ${\Delta E}_{00}$ error was selected as the optimal configuration. For the printer used in this study, the optimal parameters are as follows:

- Polyharmonic kernel: Cubic;
- Target space: CIEXYZ;
- Smoothing factor: 0.0025.

**Figure 5:**
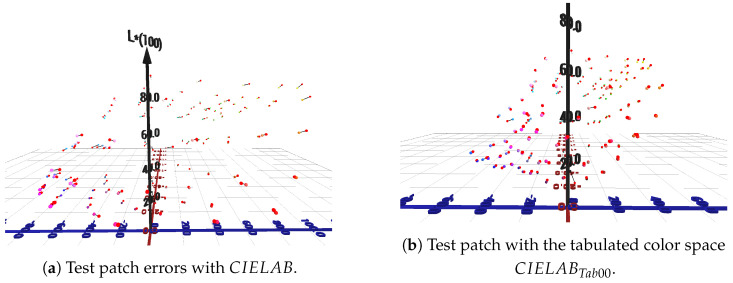
Test patch errors with CIELAB and with the tabulated color space $CIELAB_{Tab00}$. The red spheres correspond to the colors estimated by the proposed forward model.

The average, maximum errors and the 95 percentile of the corresponding forward and backward models are presented in Table 1. Figure 5 illustrates the error for each color of the test set compared to the corresponding estimated values.

After determining the optimal parameters, we generate a profile file containing all 341 RGB, CIEXYZ, and CIELAB values from the characterization as well as the model parameters.

#### 2.2.2. Gamut in *CIELAB* and *CIELAB*_*Tab*00_

**Figure 6:**
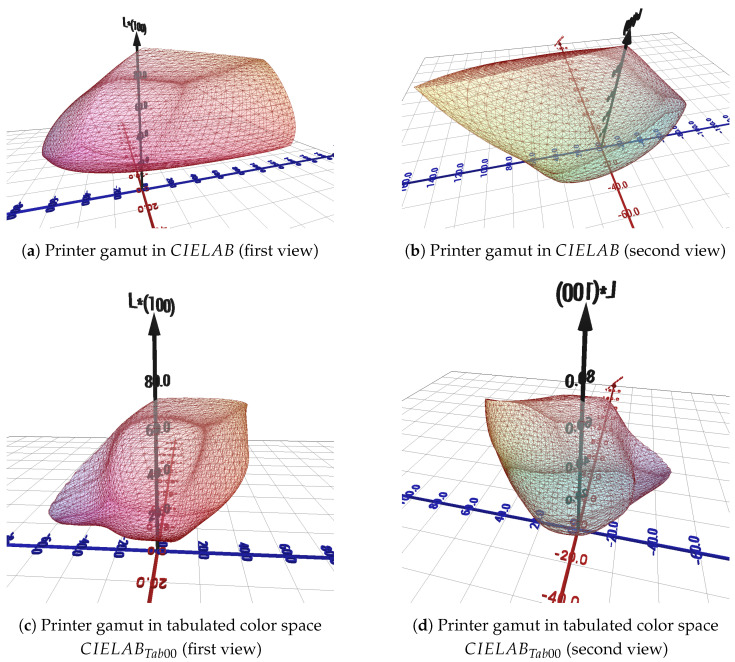
Printer gamut comparison in CIELAB and $CIELAB_{Tab00}$.

This profile allows the computation of the 3D mesh corresponding to the gamut of the printer used in CIELAB (see Figure 6a,b) as well as in the tabulated color space $CIELAB_{Tab00}$ (see Figure 6c,d), using a fine sampling of the RGB cube. As shown in Figure 6, the gamut volume of the printer is smaller in the tabulated color space $CIELAB_{Tab00}$ than in CIELAB.

The fine sampling of the RGB cube is the same as the one used to generate the set of tetrahedra that we use for the forward model. This set of tetrahedra can also be used to determine if a CIELAB and $CIELAB_{Tab00}$ triplet is within the gamut of the printer.

### 2.3. Selection of Color Reference Patches

The methodologies detailed above provide both the colorimetric and color management tools needed to select the *N* reference colors required to create the targeted pairs of color samples. Since we chose to use the full color gamut of the printer to select the reference patches, we opted for the most uniform sampling method available in the state of the art: the Kepler hexagonal stacking approach [35].

The Kepler hexagonal stack, also known as hexagonal, is particularly well suited for discretizing a 3D space due to its optimal packing efficiency and geometric properties. This arrangement forms a highly regular and repetitive structure where each sphere is surrounded by 12 other spheres at equal distances, ensuring uniform distribution with maximum packing density.

**Figure 7:**
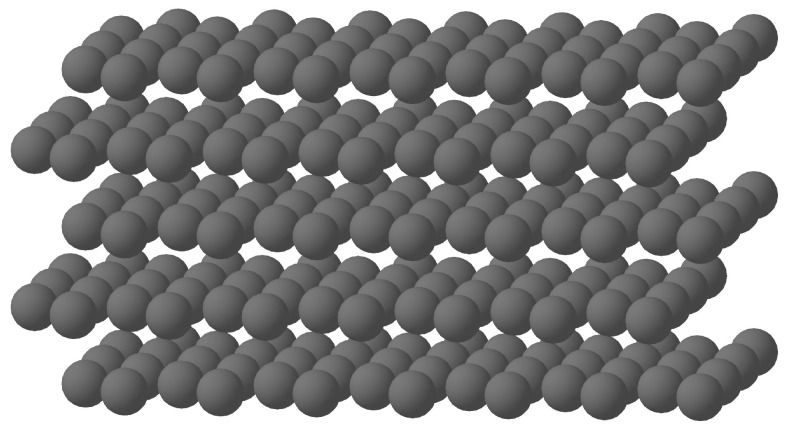
Kepler hexagonal stack.

To generate a set of colors centered on a reference color *C*, all spaced at a distance *D* within the CIELAB color space or the tabulated color space $CIELAB_{Tab00}$, we used the algorithm described in the appendix of [34] (see Figure 7).

Since the printer can only reproduce colors that fall within its gamut, we set up a simple dichotomous algorithm to determine the distance *D* at which *N* colors from a Kepler stack remain within the printer’s gamut in the tabulated color space $CIELAB_{Tab00}$. To verify whether a color lies within the gamut, we use the tetrahedral structure created for the backward model we proposed.

### 2.4. Experimental Results for 20 Reference Patches

**Figure 8:**
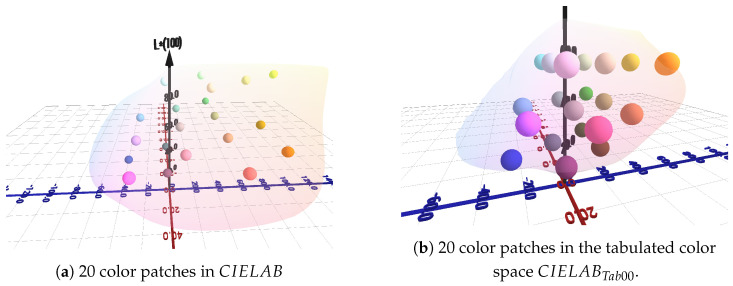
Generated color patches in CIELAB and the tabulated color space $CIELAB_{Tab00}$.

Figure 8 illustrates the results obtained when only 20 color patches are placed within the CIELAB and $CIELAB_{Tab00}$ gamuts of the printer. When the reference patches are positioned in the $CIELAB_{Tab00}$ gamut, the calculated distance is 21.29.

**Figure 9:**
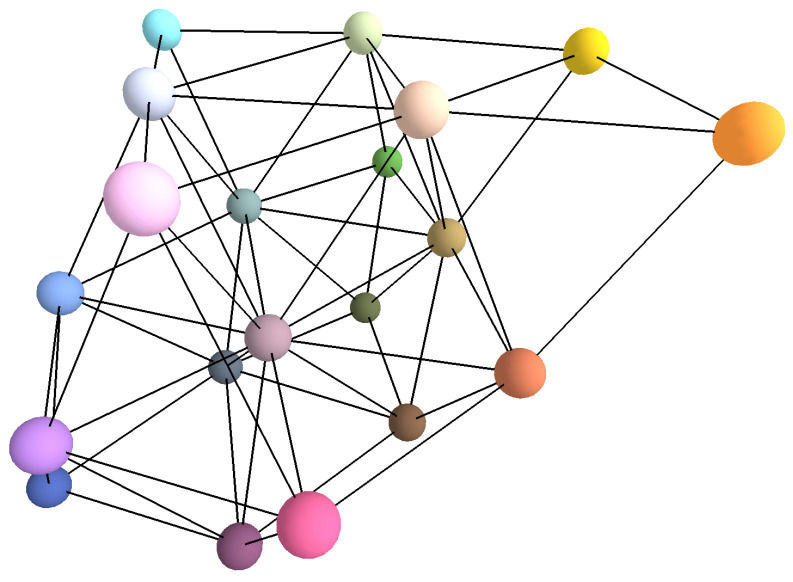
Measured grid (for 20 reference patches).

To demonstrate the validity of the proposed methodology, we used the backward model to convert the 20 reference patch colors from the tabulated color space $CIELAB_{Tab00}$ into their corresponding RGB values. These RGB colors were then printed and measured. Figure 9 shows the measured colors in the tabulated color space $CIELAB_{Tab00}$ and the corresponding hexagonal grid. The average distance within this grid is 20.91 with a minimum distance of 18.16 and a maximum distance of 24.61.

Despite the complexity of the different steps required to produce the reference patches, the proposed methodology successfully generates colors that are all equidistant within the gamut of the printer.

### 2.5. Generation of Color Pairs

We now propose to generate 10 color values for each reference color with a normal distribution of ${\Delta E}_{00}$ color distance from this reference color, which will be paired and used in the discrimination experiment that we wish to perform in the next phase of this research.

The objective was to make a compromise between the number of pairs of test samples (20 reference colors ×10 closely related colors) needed to perform a psychovisual experiment based on color discrimination and the duration of such an experiment.

Two main choices have been made in generating the color of these pairs of test patches: first, the perceptual lightness $L^{*}$ was kept the same as the reference color; second, the equidistant colors were selected randomly in the neighborhood of the corresponding reference color to avoid prioritizing any particular direction on the chromatic plane $a^{*}b^{*}$. Since we are working in the tabulated color space $CIELAB_{Tab00}$, in this space, the ${\Delta E}_{00}$ color distance is equivalent to the Euclidean distance.

To obtain a set of colors with a normal distribution of ${\Delta E}_{00}$ around the reference color, we simply sampled the tabulated color space $CIELAB_{Tab00}$ with a normal distribution of Euclidean distances to the reference colors. The direction in the $a^{*}b^{*}$ plane was selected randomly using a uniform distribution.

**Figure 10:**
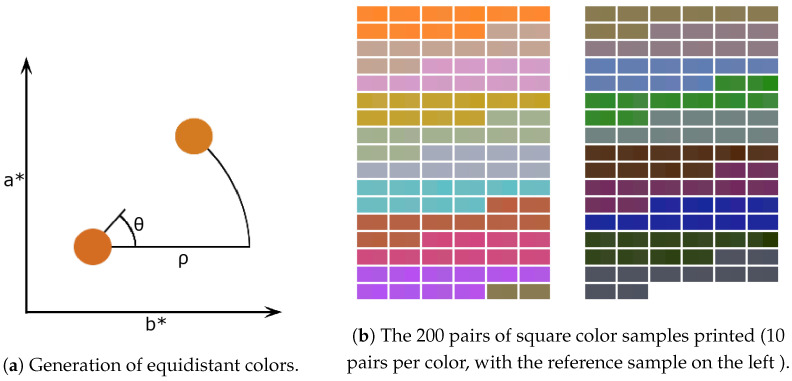
Generation of color pairs.

For a reference color $\left(L_{ref}^{*},a_{ref}^{*},b_{ref}^{*}\right)$, a closely related color $\left(L^{*},a^{*},b^{*}\right)$ is obtained as follows:

(1) $$\left\{\begin{array}{c} L^{*}=L_{ref}^{*}\\ a^{*}=a_{ref}^{*}+\rho *\sin\theta \\ b^{*}=b_{ref}^{*}+\rho *\cos\theta \end{array}\right.$$

with $\rho ∼N(\mu ,\;\sigma^{2})$ and θ∼U(0,2π). N and U are the normal and uniform distributions, respectively. The mean and standard deviation μ and σ were chosen to be set at 0.6 and 0.4, respectively, in order to obtain colors that are close to but varied from the reference color (see Figure 10a).

After printing the 200 pairs of samples (10 pairs of color per reference color, see Figure 10b), we measured the 400 color patches with the spectrophotometer. The spectrophotometer was set to perform three consecutive measurements for a given location and return the average. We made also five measurements at five different locations on each of the 400 patches (four in the corners and one in the middle) to optimize the accuracy of the measurements.

**Figure 11:**
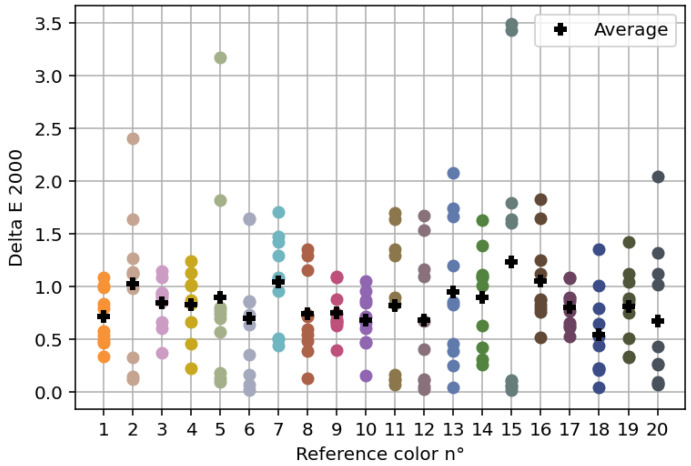
Distance between each printed color corresponding to a theoretically equidistant color and its corresponding color reference.

In Figure 11, we show the ${\Delta E}_{00}$ values for the 20 reference patches selected. The accuracy of the colors printed depends of the reference colors used (as an example, see color differences for reference colors 5 and 15). If for a reference color all ${\Delta E}_{00}$ values are lower than 1, then we can consider that there is no noticeable difference between the reference color and the printed color patches, which are then considered as equidistant to the reference color (e.g., reference colors 5 and 6). Overall, 66% of printed patches satisfied this criterion. If for a reference color, a ${\Delta E}_{00}$ value is higher than 2, then we can consider that there is a significant difference between the reference color and the printed color patch (e.g., for reference color 15, two color patches are concerned). Very few printed patches were concerned (3%), a few more had a ${\Delta E}_{00}$ between 1.5 and 2 (8.5%). This demonstrates that the method proposed to print equidistant colors with small color differences is efficient and can be used to produce pairs of color patches that could be used to perform the color discrimination experiment. Only 6 of the printed patches have a ${\Delta E}_{00}$ higher than 2 and can thus be considered as outliers. We decided to remove them from the set as they are not adapted for a color discrimination experiment, leaving us with a total of 194 color pairs.

### 2.6. Printing Stability

To assess the stability of the prints over time, we produced a page containing 20 color patches (one patch per reference color). After a drying period of one week, these patches were measured weekly over one month. No significant changes were observed in the measurements, all of which remained within the tolerance range of the spectrocolorimeter (with an average ${\Delta E}_{00}$ error of 0.2). However, we plan to re-measure the color patches before starting the psychophysical experiments.

## 3. Methods Used for LED Light Stabilization

The next step of this research work will be to set up a psychovisual experiment that will allow us to evaluate the ability of human observers to discern small color distances under a given lighting. In such context, a perfectly stabilized light is essential. In this section, we detail a method for stabilizing multi-LED illuminants that we have developed specifically for the experimental protocol described in Section 4.

### 3.1. Technical Specifications

The spectrally tunable LED panel used in this work is a Dittosizer light player from Telelumen (see Table 2 for its main characteristics). The panel was suspended from the ceiling of a 250 × 250 cm light booth cabin with white walls. Overall, 19 of the 24 available channels were retained for characterization purposes. Both ultraviolet (UV) and infrared (IR) channels were excluded as they fall outside the visible range. In addition, one channel was omitted due to its non-linear behavior in comparison to its drive level.

**Figure 12:**
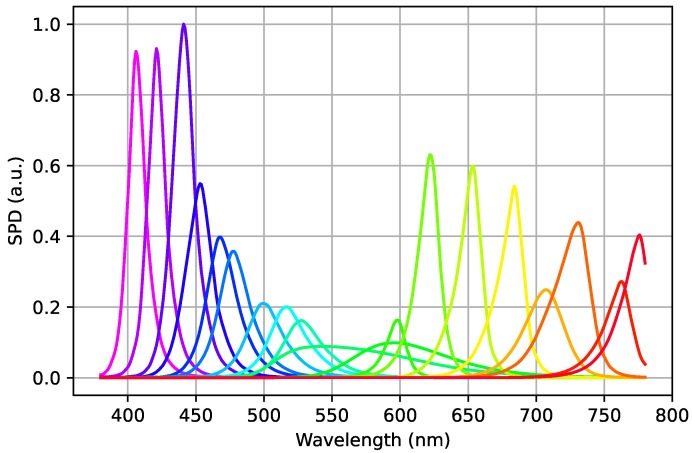
Spectral Power Distribution of the 19 selected channels within the visible range of the Telelumen LED panel measured with a spectral resolution of 5 nm and interpolated to 1 nm with akima interpolation.

The measurements of the Spectral Power Distributions (SPDs) of light sources were performed using a JETI spectraval 1511 spectroradiometer. The characteristics of this spectroradiometer are summarized in Table 3. The measured SPDs of the 24 LEDs in the Telelumen panel are shown in Figure 12. The range selected for the measurement was from 380 nm to 780 nm with a spectral resolution of 5 nm.

The tunable LED panel and the spectroradiometer were connected to a PC (i7, 32GB RAM) using their respective Application Programming Interfaces (APIs), Python 3.10 and the Luxpy library [36]. The Telelumen panel can be controlled by directly sending an array of 24 float values between 0 and 1 representing the drive levels for each channel. The Telelumen panel API can also be used to obtain readings from internal temperature sensors.

### 3.2. Characterization Procedure

**Figure 13:**
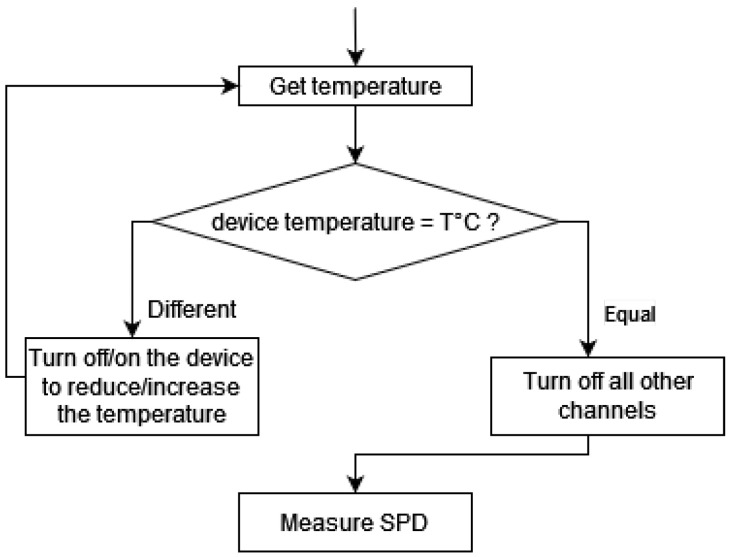
Flowchart of the characterization procedure. © Vernet et al. [1].

As mentioned previously, the objectives of the characterization were both to evaluate the impact of temperature on the light output of a multi-channel LED lighting system and to identify the difficulties linked to the stabilisation over time the emitted spectrum. A method was devised in [1] to consistently measure the light output at different device temperatures. As the 19 individual channels chosen are entirely independent, the characterization of the Telelumen LED panel was carried out by measuring the luminous flux of each channel separately. This was achieved across 100 different drive levels and over 15 temperature settings ranging from 30 °C to 45 °C. To regulate the temperature of the device, all LEDs were turned on until the device reached the desired temperature just before each measurement. The detailed procedure for characterization is outlined in Figure 13. Throughout the measurements, the spectroradiometer was positioned directly under the LED panel at a distance of approximately 55 cm.

### 3.3. Characterization Results

The measurement values obtained at the minimum (30 °C) and the maximum (45 °C) temperature of the heatsink are summarized in Table 4. The measurement results clearly show that temperature affects all channels to varying degrees. Channels V1 to B1 are less sensitive and have reduced loss of luminous efficacy and no perceptible spectral shift. B2 to G2 have a more pronounced loss of luminous efficacy as well as a slight spectral shift. Channels L to FR3 exhibit significant intensity loss, reaching a maximum of 30% on channel R1, which is accompanied by a significant spectral shift toward the longer wavelengths of the visible range. This shift is gradual with increasing of the temperature (noted as “grad” in Table 4). Additionally, peak amplitude was measured to check its proportionality with the drive level at different temperatures. It was confirmed that all selected channels exhibit almost linear properties, although the slope decreases with decreasing luminous efficacy.

### 3.4. Theoretical Optimization

In theory, with a comprehensive characterization of the device, as discussed in the previous section, and a detailed understanding of all relevant parameters, it would be possible to accurately predict the input drive levels required to replicate a target spectrum.

Since the output of an LED device is the combined emission of its individual channels, the precise output can be predicted using the following calculation:

(2) $$\forall \lambda \in \left[380,780\right],S_{T,I}\left(\lambda \right)=\sum_{i=0}^{L-1}S_{T^{i},I^{i}}^{i}\left(\lambda \right)$$

where λ is the wavelength ranging from 380 to 780 nm, *L* is the number of channels and $S_{T^{i},I^{i}}^{i}$ is the spectral distribution of the $i^{th}$ channel at temperature $T^{i}$ and drive level $I^{i}$.

Different types of minimization can then be performed. Both Mean Squared Error (MSE) and Mean Absolute Error (MAE) were evaluated. No significant differences were noticed in stabilization performance. Consequently, MSE was selected for this task:

(3) $$I^{new}=\min_{I}\int_{380}^{780}{\left(Ta\left(\lambda \right)-\sum_{i=0}^{L-1}S_{T^{i},I^{i}}^{i}\left(\lambda \right)\right)}^{2}d\lambda$$

with Ta the target SPD to match.

However, in absence of a full device characterization, the values of $S_{T^{i},I^{i}}^{i}\left(\lambda \right)$ are not directly accessible. Therefore, we proposed in [1] an approximation method based on measurements taken at the maximum drive level at a single temperature.

### 3.5. Approximation of the Impact of the Temperature

It is possible to approximate the output of an LED at any drive level using the spectrum of the output of the maximum drive level with a linear approximation [37]:

(4) $$S_{T_{0},I_{0}}\left(\lambda \right)=I_{0}S_{T_{0},1}\left(\lambda \right)$$

with $S_{T_{0},I_{0}}$ the SPD of an LED at the temperature $T_{0}$ and drive level $I_{0}$, and $S_{T_{0},1}$ the SPD at its maximum drive level.

The main limitation of this approximation is its viability only under constant temperature conditions.

The impact of temperature on channel output can be represented by incorporating an intensity offset dI and a wavelength offset dλ such as

$$S_{T_{1},I_{0}}^{i}\left(\lambda \right)=\left(I_{0}^{i}+dI^{i}\right)S_{T_{0},1}^{i}\left(\lambda +d\lambda^{i}\right)$$

We can obtain these deviations by minimizing the MSE criterion with a single measurement of the device output.

(5) $$\left(dI_{min},d\lambda_{min}\right)=\min_{dI,d\lambda }\int_{380}^{780}{\left(M\left(\lambda \right)-\sum_{i=0}^{L-1}\left(I^{i}+dI^{i}\right)S_{T_{0},1}^{i}\left(\lambda +d\lambda^{i}\right)\right)}^{2}d\lambda$$

with *M* the measured spectrum.

### 3.6. Optimization of the Drive Levels

Ultimately, to determine the updated drive levels, we propose to simply substitute the theoretical values in Equation (3) with the approximation obtained with Equation (5):

(6) $$I^{new}=\min_{I}\int_{380}^{780}{\left(Ta\left(\lambda \right)-\sum_{i=0}^{L-1}\left(I^{i}+dI_{min}^{i}\right)S_{T_{0},1}^{i}\left(\lambda +d\lambda_{min}^{i}\right)\right)}^{2}d\lambda$$

with Ta the target spectrum.

### 3.7. Stabilization Results

**Figure 14:**
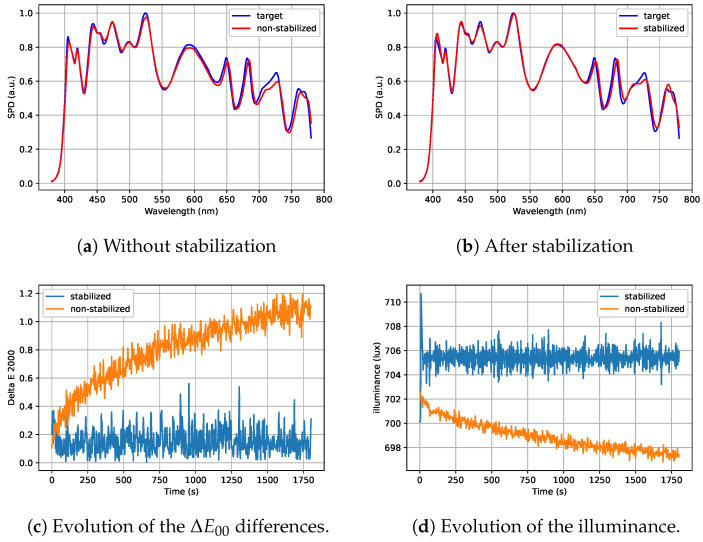
Comparison of the output with (blue chart) and without (orange chart) stabilization for illuminant D65.

**Figure 15:**
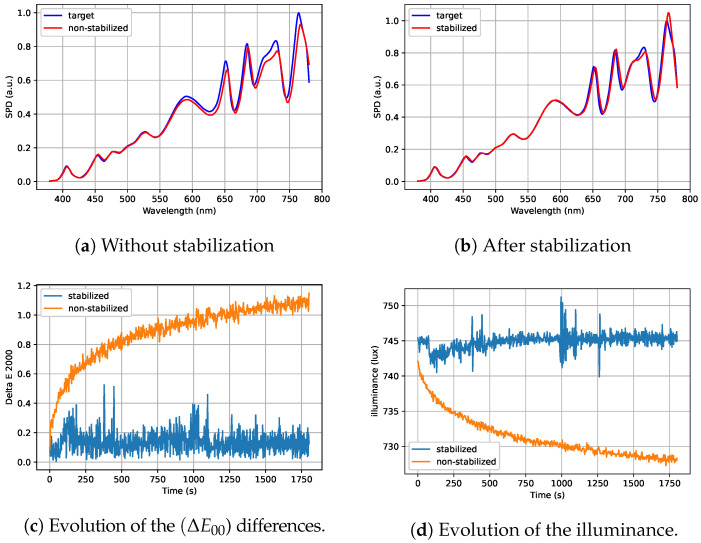
Comparison of the output with (blue chart) and without (orange chart) stabilization for illuminant A.

The algorithm was validated using several Spectral Power Distributions (SPDs). The best performance was observed when all channels were active, as this provides greater flexibility for corrections. Figure 14 and Figure 15 show two examples of SPDs close to the illuminant D65 and illuminant A, respectively. Figure 14a and Figure 15a highlight a significant spectral shift away from the target when the light output is not stabilized. This shift is particularly noticeable in the channels corresponding to longer wavelengths as they are more sensitive to heat. In contrast, Figure 14b and Figure 15b illustrate how the stabilization algorithm improves the spectral match between the light output and the target spectrum. This improvement in spectral matching comes with a decrease in color differences relative to the target, as shown in Figure 14c and Figure 15c. In these two examples, the not stabilized light output exhibits a ${\Delta E}_{00}$ color difference exceeding 1, which corresponds to a perceptible color difference. With stabilization, the ${\Delta E}_{00}$ is reduced to a value below 0.4, making the color difference imperceptible. Additionnally, the illuminance is stabilized, as illustrated in Figure 14d and Figure 15d.

## 4. Protocol Proposed for the Color Discrimination Experiment

The main constraint in designing this experiment was to ensure simplicity for future participants. The proposed protocol requires participants to observe a pair of color patches and indicate whether they can distinguish between the two colors. Such an approach allows participants to focus solely on the task of color discrimination. By eliminating the need to pre-order the samples, the protocol significantly reduces the duration of the experiment and removes the need for a complex sample-feeding system. To achieve this, the color pairs are placed on a thick cardboard support covered with neutral gray paper for easy handling. An ArUco code is printed on the reverse side of the support to identify the color pair being examined by the participant.

The experimental setup was designed to be as simple as possible: a box containing all pairs of samples not yet examined; a holder for the pair currently under examination; and a box for discarding pairs that have already been examined. Additionally, a three-button keypad is connected to a computer equipped with a camera to read the ArUco codes, allowing the recording of participants’ responses.

### 4.1. Experimental Setup

The light booth used for the color discrimination experiment consists of a 2.5 m × 2.5 m × 2.5 m room with three white walls at the front and a black curtain at the back to allow participants to enter. The light booth is equipped with the Telelumen LED panel described in Section 3.1. A JETI spectroradiometer (also described in Section 3.1) is installed in the booth. To prevent interference with the experiment, the spectroradiometer is covered with a matte neutral-colored material. The chair is black, fully adjustable, and includes both armrests and a headrest to ensure participant comfort.

**Figure 16:**
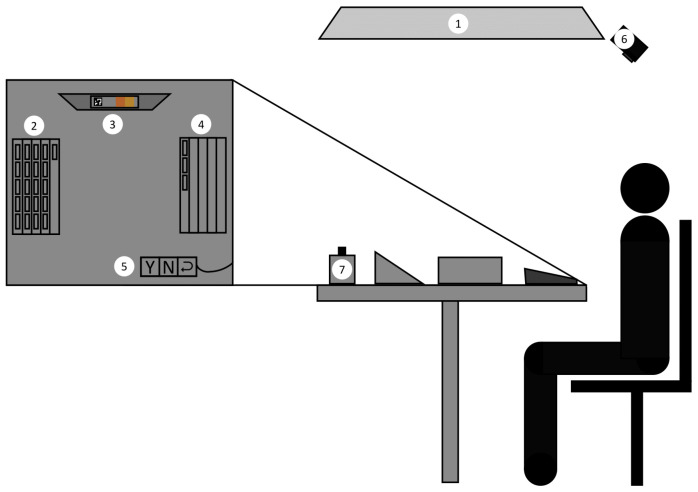
Experimental setup: (1) LED panel, (2) box of samples not yet examined, (3) sample support, (4) box for discarded samples, (5) three buttons keypad, (6) camera, (7) spectroradiometer.

The table measures 70 × 70 cm, has an adjustable height and is covered with matte neutral-gray material with a lightness value of 40% in regard to a white patch. Such a value corresponds to a medium-dark gray, which minimizes adaptation effects that would occur with too bright (white) or too dark (black) backgrounds [38]. On the left side of the table, a matte neutral-gray box is placed to hold pairs of samples not yet examined. The samples will be arranged vertically within the box to hide their color and save space. At the center of the table, a small support angled at approximately ∼45° is used to hold the pair of samples currently being analyzed. On the right side, another box is provided for discarding pairs that have already been analyzed. A keypad will be placed on the table, which is covered with matte neutral-gray material to minimize distraction, exposing only three buttons for the options “yes”, “no”, and “change the last answer”. Finally, a camera is mounted at the top of the stand at an angle to record the order in which samples are examined. Figure 16 illustrates the complete experimental setup.

### 4.2. Experiment Samples

For simplicity of implementation of the protocol, the two patches constituting the pairs are squares of size 1.4 cm × 1.4 cm, which corresponds to a 2° angle at 40 cm. The paper used for printing the patches is a matte photo paper with a paper weight of 189 g/m^2^, allowing for vibrant color as well as avoiding ripples of the paper due to the ink. The two patches of color are printed brought together and bonded with each other in order to avoid the contrast effect that would arise with a gray band between them. The color difference between adjacent patches should be small enough so that the Mach bands effect is negligible.

**Figure 17:**
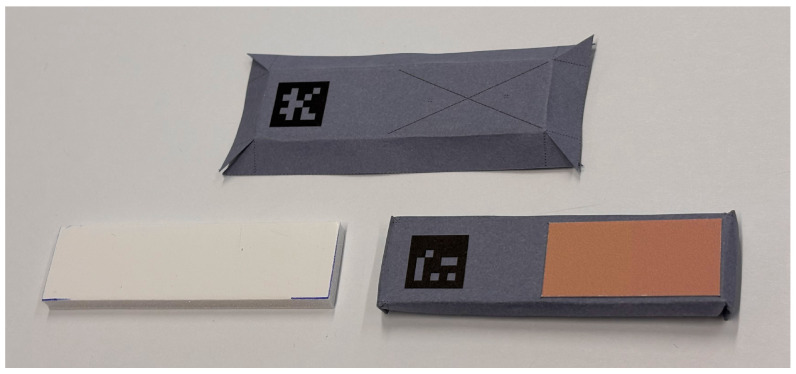
Production of the experimental samples.

To simplify the task of handling the samples during the experiment, the color pairs are then glued to a thick cardboard support. This support is covered on all sides with the same neutral gray paper as the table. On one side, the location of the pair is marked with a cross to ensure that the position is the same for all pairs of patches, and on the other side, an ArUco marker is printed to automatically process the number of the sample being examined by the participant. The steps for the production of the experimental samples are shown in Figure 17. The choice of the ArUco was made after considering three options: numbers and optical character recognition (OCR); QR codes; and ArUco markers. OCR was discarded to avoid any readable characters for the participant. The choice of ArUco over QR codes was made after several tests showing that ArUco is more robust at a further distance, at a given viewing angle, and with non-white paper.

### 4.3. Experiment Workflow

Before carrying out the experiment, several preliminary steps must be completed. First, participants must be screened for color vision deficiencies using an online Farnsworth–Munsell 100 hue test. Following this, instructions will be provided both orally and in written form. Participants will be shown a sample (not included in the actual experiment) to familiarize them with the procedure and demonstrate proper handling to avoid touching the surface of the color patches. Afterward, participants will have an opportunity to ask any questions they may have.

The experiment workflow is managed by a series of Python scripts that coordinate the lighting system, the spectroradiometer used for light stabilization, the camera and the keypad used to record the responses of the participants.

**Figure 18:**
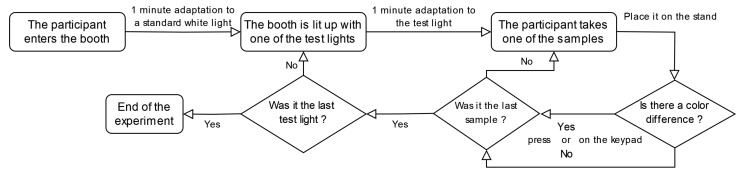
Experiment workflow.

This workflow is summarized in Figure 18.

### 4.4. Experiments and Protocol Validation

This protocol will be used for two complementary experiments. The first, with 194 color pairs and a limited number of subjects (∼20 observers), will allow us to validate and, if necessary, adjust the experimental protocol defined. This will help us to define more precisely the number of reference patches, the number of color pairs to be produced, and the number of subjects required for the final experiment.

The second experiment will be conducted with subjects equipped with low-vision simulation goggles in the continuity of the work that has already been conducted on optimizing light for low-vision people. This second step will greatly benefit from the first experiment as it will have both a bigger number of participants and lights to test.

## 5. Critical Analysis and Perspectives of This Work

In this section, we will conduct a critical analysis of the two methods we have proposed in the framework of this paper and, for each of them, we will present some perspectives that they offer.

### 5.1. Creation of Color Patches

The color management method proposed in this paper has so far been validated on a single printer. To fully validate the approach, we will need to apply it to other printers. However, the results obtained are already ideal for the psychophysical experiments we are preparing to launch. The validation tests will focus on printers with similar characteristics, such as those offering a wide range of inks and print papers suitable for this purpose.

Once these validations will be completed, the method could be applied to creating custom color charts for camera calibration. In [34], we show that for the forward model based on polyharmonic splines, it is preferable that a given number of patches have uniformly placed colors in the target color space. Using this method, we can directly design and print color charts tailored for calibrating image sensors. We can also consider creating supplementary color patches to complement those already present in standard color charts such as the xRite ColorChecker Digital SG.

### 5.2. LED Light Stabilization

The proposed light stabilization algorithm has been tested only with the Telelumen LED panel and the JETI 1511 spectroradiometer. However, the Python script was designed with flexibility in mind and can be easily adapted for use with any equipment that provides an accessible API.

Optimizing the code could improve its performance, as the algorithm currently requires up to 1 s per iteration (on a computer equipped with an Intel i7-12700H processor). The script is currently compatible with Python 3.10 due to library constraints. However, upgrading to the newer Python 3.13 version is under consideration to benefit from enhanced multithreading capabilities. The most time-consuming step is the light measurement process, and efforts should be made to reduce this time, potentially by changing the position of the spectroradiometer. It is worth noting that only one spectroradiometer was available for this work, which may have influenced the efficiency of the setup.

A potential limitation of the algorithm lies in its minimization function used to optimize the panel drive levels, which only takes into account the spectral matching to the target. While this approach ensures a stable output and closely matches the target illuminance, color accuracy could be further improved by including a cost function that balances spectral similarity with colorimetric accuracy.

For computer vision applications, our stabilization technology can be integrated into image or video capture systems. This results in colorimetrically stable images that provide more accurate results for computer vision tasks such as image classification, object detection, and tracking.

## 6. Conclusions

In this paper, we introduced a comprehensive experimental protocol for evaluating color discrimination under controlled LED lighting conditions. Our approach combines innovative techniques for generating color samples and stabilizing LED lighting. Addressing critical challenges in color discrimination experiments, we presented an innovative method for producing custom color patches using a colorimetrically calibrated inkjet printer and an advanced stabilization method for multi-channel LED lighting systems. These techniques address two major issues in color discrimination experiments: ensuring accurate color reproduction for creating tailored color patches and maintaining a stable lighting environment.

By integrating a tabulated color space with appropriate sampling techniques, we successfully created customized color pairs based on 20 equidistant reference colors within the gamut of our target printer, which is suitable for studying color discrimination. Additionally, our LED stabilization algorithm which leverages spectral characterization and drive-level optimization ensures consistent illumination and minimizes thermal and spectral variability.

The methods developed in this study not only improve the technical capabilities of psychophysical experiments but also open up possibilities for practical applications, such as the development of customized color charts for imaging system calibration and the improvement of lighting devices for both visually impaired and sighted people. This work provides a strong foundation for a better understanding in color perception and the need of controlled lighting in vision research.

## Figures and Tables

**Table 1:** Comparison of errors using **CIEDE76** and **CIEDE00** metrics.

Metric	CIEDE76	CIEDE00
Forward model		
ΔE—Mean	1.55277	0.825391
ΔE—Max	7.01033	5.55854
ΔE—95 percentile	3.57306	1.65521
Backward model		
ΔRGB—Mean	0.0305036	0.0302672
ΔRGB—Max	0.150043	0.150419
ΔRGB—95 percentile	0.0981143	0.0959103

**Table 2:** Characteristics of the Telelumen Dittosizer light player.

Size	602 × 602 mm
Power consumption	∼100 W
N° of channels	24 (365–940 nm)
Max luminous output	∼5000 lumen
Precision	250:1

**Table 3:** Characteristics of the JETI spectraval 1511 spectroradiometer.

Spectral range	350…1000 nm
Spectral resolution	5 nm
Measuring range	0.2…140,000 cd/m^2^

**Table 4:** Characterization of the 19 channels of the Telelumen LED panel in the visible range. © Vernet et al. [1] (a nomenclature provided by TELELUMEN is assigned to each LED: V1, V2, … , FR2, FR3).

	V1	V2	RB1	RB2	B1	B2	C	G1	G2	L
Peak wavelength (nm)	406	421	441	454	468	478	500	517	528	544
Amplitude attenuation	7%	6%	4%	3%	4%	5%	16%	1%	15%	2%
Peak shift (nm)	no	no	no	no	no	<10	<10	<10	<10	grad
	PC-A	OR	R1	R2	DR1	DR2	FR1	FR2	FR3	
Peak wavelength (nm)	594	624	636	654	686	708	732	765	777	
Amplitude attenuation	8%	27%	30%	28%	6%	21%	28%	10%	7%	
Peak shift	grad	grad	grad	grad	grad	grad	grad	grad	grad	

## Data Availability

The original contributions presented in this study are included in the article. Further inquiries can be directed to the corresponding author.
